# Allergic sensitization: host-immune factors

**DOI:** 10.1186/2045-7022-4-12

**Published:** 2014-04-15

**Authors:** Ronald van Ree, Lone Hummelshøj, Maud Plantinga, Lars K Poulsen, Emily Swindle

**Affiliations:** 1 Departments of Experimental Immunology and Otorhinolaryngology, Academic Medical Center, University of Amsterdam, Meibergdreef 9, Room K0-130, 1105 AZ, Amsterdam, The Netherlands; 2 Laboratorium for Medicinsk Allergologi, Copenhagen University Hospital at Gentofte, Niels Andersens Vej 65, DK-2900 Hellerup, Denmark; 3 Laboratory of Translational Immunology, University Medical Center Utrecht, Heidelberglaan 100, 3584 CX, Utrecht, Netherlands; 4 Allergy Clinic, Copenhagen University Hospital at Gentofte, Niels Andersens Vej 65, DK-2900 Hellerup, Denmark; 5 Academic Unit of Clinical and Experimental Sciences, Faculty of Medicine, University of Southampton, Tremona Road, Southampton SO16 6YD, United Kingdom

**Keywords:** Allergic sensitization, Protein, Allergic inflammation, Food allergy, Endogenous allergen

## Abstract

Allergic sensitization is the outcome of a complex interplay between the allergen and the host in a given environmental context. The first barrier encountered by an allergen on its way to sensitization is the mucosal epithelial layer. Allergic inflammatory diseases are accompanied by increased permeability of the epithelium, which is more susceptible to environmental triggers. Allergens and co-factors from the environment interact with innate immune receptors, such as Toll-like and protease-activated receptors on epithelial cells, stimulating them to produce cytokines that drive T-helper 2-like adaptive immunity in allergy-prone individuals. In this milieu, the next cells interacting with allergens are the dendritic cells lying just underneath the epithelium: plasmacytoid DCs, two types of conventional DCs (CD11b + and CD11b-), and monocyte-derived DCs. It is now becoming clear that CD11b+, cDCs, and moDCs are the inflammatory DCs that instruct naïve T cells to become Th2 cells. The simple paradigm of non-overlapping stable Th1 and Th2 subsets of T-helper cells is now rapidly being replaced by that of a more complex spectrum of different Th cells that together drive or control different aspects of allergic inflammation and display more plasticity in their cytokine profiles. At present, these include Th9, Th17, Th22, and Treg, in addition to Th1 and Th2. The spectrum of co-stimulatory signals coming from DCs determines which subset-characteristics will dominate. When IL-4 and/or IL-13 play a dominant role, B cells switch to IgE-production, a process that is more effective at young age. IgE-producing plasma cells have been shown to be long-lived, hiding in the bone-marrow or inflammatory tissues where they cannot easily be targeted by therapeutic intervention. Allergic sensitization is a complex interplay between the allergen in its environmental context and the tendency of the host’s innate and adaptive immune cells to be skewed towards allergic inflammation. These data and findings were presented at a 2012 international symposium in Prague organized by the Protein Allergenicity Technical Committee of the International Life Sciences Institute’s Health and Environmental Sciences Institute.

## Introduction

In April 2012, an international symposium titled “Sensitizing Properties of Proteins” was held in Prague, Czech Republic, bringing together over 70 scientists from academia, government, and industry. The purpose of the symposium, organized by the Protein Allergenicity Technical Committee (PATC) of the International Life Sciences Institute’s (ILSI) Health and Environmental Sciences Institute (HESI), was to present data on the current state of the science regarding the sensitizing properties of proteins in relation to food allergy [1,2]. Host-immune factors are the focus of this paper.

Allergic sensitization induced by exogenous allergenic molecules is the result of exposure of organs of the human body to such molecules. In this interplay, intrinsic properties of the exogenous proteins and environmental co-factors certainly play a role, but host-immune factors are of crucial importance to explain why every individual exposed to such an allergen does not develop an allergy. Predisposition for developing allergy is the result of a complex multifactorial interplay of genes and environment. To understand the immunobiological mechanism of sensitization to allergens, their interaction with relevant structural and immune cells during mucosal exposure and entry is of the utmost importance. These cells include cells of the first physical barrier, the epithelial cells; professional antigen-presenting cells, mainly dendritic cells (DCs); and T-helper cells and B cells. Together these cells shape the process of allergic sensitization. An overview is given here about their respective roles in the initiation of allergic inflammation.

### The role of the epithelium in sensitization

The epithelium is a membranous tissue which covers the external surface and lines the internal compartments of organs within the body. At these sites, the role of the epithelium is to form barriers which define boundaries and prevent the unrestricted exchange of materials. Depending on the type of epithelium, it will serve specialized functions relevant to the organ in which it is found. For example in the conducting airways, the epithelium allows the passage of air to the gas exchange regions of the lung and forms a physical, chemical, and immunological barrier.

### Physical barrier

The physical epithelial barrier is composed of a polarized epithelium which is selectively permeable to ions and macromolecules due to the presence of receptors, transporters, and tight junctions (TJ). Receptors and transporters control the airway surface liquid (ASL) volume which hydrates the mucus layer and provides optimal conditions for the beating of cilia. Both the mucus layer and the beating of cilia (together referred to as the mucociliary escalator) are essential for the efficient removal of particles and proteins. The epithelial TJs regulate the permeability of the epithelial barrier to solutes based on their size and charge and, as a result, also contribute to ASL volume. They are a complex structure of several proteins including occludins, claudins, and zona occludins and are expressed on the most apical surface of individual epithelial cells (ECs) within the epithelial barrier. They perform fence and gate functions, are dynamic complexes, and alterations in both the distribution and levels of TJ proteins affect the integrity of the epithelial barrier [3]. The chemical epithelial barrier contains secretions including mucus (primarily mucins), cytoprotective proteins (anti-oxidants) and host defense molecules (β-defensins) which trap and inactivate inhaled particles and pathogens. These are then cleared from the airways via the mucociliary escalator.

### Immunological barrier

The immunological epithelial barrier of the airways performs immune surveillance through the expression of innate immune receptors, including pathogen recognition receptors (PRRs). In response to bacteria, viruses, and fungi, EC activation via PRRs leads to the release of an array of mediators and damage-associated molecular patterns which exert an effect on underlying resident immune cells to induce an inflammatory response. In this way, epithelial cells can control the function of many immune cells in the underlying mesenchyme, including DCs, mast cells (MCs), eosinophils and T cells involved in both innate and adaptive immunity (reviewed by Swindle et al. [4]).

### Impact of foreign proteins on barrier function

There are various mechanisms by which proteins can penetrate the epithelium through alterations in components of the physical, chemical and immunological epithelial barrier. Allergens such as house dust mites have protease activity which can alter the physical barrier by directly interacting with TJ proteins [5,6]. Pathogens (viruses, bacteria, and fungi) and environmental stresses (cigarette smoke) are known to disrupt the physical barrier [7,8]. Activation of the chemical barrier can also be induced by environmental factors, as well as host-derived cytokines, such as IL-13, which increases mucus production [9]. Furthermore, the direct action of allergens via protease-activated receptors (PARs) and pathogens via PRRs cause activation of the immunological barrier through the release of mediators which induce inflammation and recruitment of immune cells.

The activation of the epithelial barrier by proteins is particularly important when thinking about diseases of the airways such as asthma where these factors are known to contribute and exacerbate the disease. Asthma is an inflammatory disease of the conducting airways with pathological features of inflammation and airway remodeling. The airways of asthmatics undergo distinct and functional changes leading to non-specific bronchoconstriction and airway obstruction and respond too easily and spontaneously to environmental factors. The epithelial barrier in asthmatics is disrupted and undergoes cycles of damage and repair which, in the presence of underlying inflammation, perpetuates the disease [10]. Also, airway epithelial cells from asthmatic subjects are more sensitive to environmental triggers including cigarette smoke [11] and viruses [12] in terms of both barrier disruption and an innate defect in the release of mediators. Furthermore, when the epithelial barrier is not disrupted, DCs can sample allergens without disrupting the epithelial barrier through the expression of TJ proteins [13]. The epithelial immunological barrier can control the adaptive immune response to allergens. For example, in a mouse model of asthma, structural cells were shown to be pivotal for controlling DC function and the development of asthma [14]. Hence, in asthmatics, alterations in the epithelial barrier to environmental triggers would allow increased penetration of proteins and allergens to the underlying mesenchyme increasing the likelihood of activation of both innate and adaptive immunity.

### *In vitro* model of the epithelial barrier

There are various *in vitro* models which can be used to investigate the potential of proteins to modulate the epithelial barrier. These vary in complexity from EC lines to primary ECs derived from healthy and asthmatic subjects. These cells can be grown on porous membranes which cause their polarization and differentiation into a multilayered epithelial barrier containing mucus-producing goblet cells, ciliated ECs, and polarized TJ protein expression. More complex models include incorporating underlying structural cells (fibroblasts) and immune cells (DCs, MCs, and macrophages) to study the interaction of different cell types in asthma (reviewed in Swindle et al. [15]). Furthermore, the epithelial barrier can be monitored by transepithelial resistance measurements using chopstick electrodes to determine ion permeability or incubated apically with fluorescently labeled proteins (FITC-dextran) of different sizes to determine paracellular permeability into the basal compartment [11]. A similar fluorescent method can also be used to determine alterations in ASL volume [16]. Alterations in TJ proteins in these cultures can be monitored by determining the distribution of TJ using immunofluorescence and analysis by fluorescent microscopy.

In summary, the epithelial barrier is integral to restricting the free passage of proteins and ions to the underlying tissue, and comprises a physical, chemical, and immunological barrier. There are mechanisms by which proteins and other substances can penetrate this barrier and mount an immune response, and there are various *in vitro* models which can be used to test the potential of proteins to disrupt the epithelial barrier.

## Dendritic cells: subtypes and how they are activated

### Role of DCs in T-helper cell polarization

Lung DCs control T-helper cell polarization towards a Th1, Th2, or Th17 response, or conversely, prevent harmful immune responses to inhaled antigen via the induction of regulatory T cells.

DCs control immune responses to a variety of inhaled antigens, including allergens and viruses. It has been reported that DC ablation during the sensitization effector phases of the allergic response abolished typical features of asthma, like eosinophilic influx, Th2 cytokine production, or airway hyper-responsiveness (AHR) [17]. However, in response to influenza, DC depletion led to increased virus titres and a reduced number of virus-specific CD8 T cells [18]. These data indicate that although depleting DCs might be beneficial in the treatment for asthma, such strategy would not be safe and might impede host-immune responses to pathogens. Therefore, trying to unravel a specific role for different DC subsets in specific diseases and trying to target such subsets might represent a better alternative.

### DC subsets

**Figure 1:**
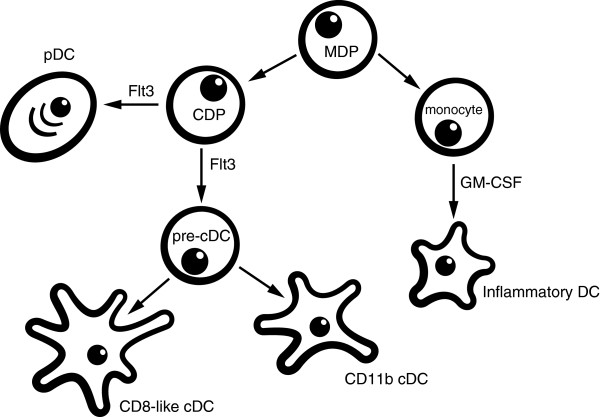
Dendritic cell (DC) subsets.

DCs can be divided into different subsets according to their expression profile and location [19]. Until now, four major populations are described in the lung, of which some are originating from different precursors (Figure 1). The generation of DCs starts in the bone marrow, where macrophage and DC precursors (MDPs) develop into common DC precursors (CDPs). From this end, plasmacytoid DCs (pDCs) separate in their developmental stage from conventional DCs (cDCs), because these CDPs are precursors for pDCs (subset 1) as well as pre-DCs, which develop into cDCs [20]. The cDCs in the lung can be further discriminated by their CD11b expression into CD11b^+^ (subset2) and CD11b^-^ DCs, of which the latter expresses CD103 and langerin (subset3). These and other reports suggest that CD8^+^ splenic and CD103^+^ DCs in the dermis, lung, gut, and liver are related to each other. Interestingly, it is shown that both CD103^+^ dermal DCs and CD8^+^ spleen DCs are specialized in antigen cross-presentation, and a similar effect is shown for the CD103^+^ subset in the lung [21,22].

In contrast, under inflammatory conditions, cytokines and chemokines, which attract monocytes, are produced [23]. These monocytes can develop into CD11b^+^ inflammatory DCs (subset 4). The inflammatory DCs have been shown to express ly6C, a marker remnant from their monocytic descent, but, after differentiation, they start to lose Ly6C expression, and as a consequence cannot be discriminated from the CD11b^+^ cDCs. Therefore, an additional marker is needed to discriminate the different subsets after recruitment by, for example, house dust mite (HDM). Recently, investigators have reported that, in the lung, these recruited CD11b^+^ cells also express FcϵR1 [24]. This suggests that expression of the high affinity IgE receptor might be useful to identify monocyte-derived inflammatory DCs (moDCs) in the lungs.

### Mouse model to study the role of DC subsets

The exact role of these different DC populations remains unclear. Investigators have previously shown that HDM induces Th2 immunity and allergic asthma through Toll-like receptor (TLR)-4 triggering on epithelial cells. In turn, activated epithelial cells produce several mediators that instruct DCs in the lung to induce Th2 responses [25]. To be able to study lung DC activation in more detail, a transgenic mouse line was developed, which expresses the beta chain of a TCR specific for a peptide of Der p 1 on CD4 T cells. This tool allows the dissection of the specific contribution of each lung DC population to HDM-induced allergic airway sensitization. To test the role of the cDCs and moDCs in Th2 sensitization, these cells were sorted from the mediastinal lymph node (MLN) of HDM-sensitized mice. These cells were adoptively transferred intratracheally into recipients, which were challenged with HDM allergens a week later. Strikingly, the adoptive transfer of only a few CD11b^+^ cDCs or moDCs was able to induce Th2 sensitization to HDM, as assessed by an eosinophilic influx to the lung, and the Th2 cytokine profile in the supernatant of re-stimulated MLN cells. Surprisingly, the transfer of CD103^+^ cDCs failed to induce any features of allergic airway inflammation [26]. These results suggest that CD11b^+^ DCs (cDC or moDC) can induce HDM-induced Th2-associated allergic airway inflammation. Why CD103^+^ cDCs fail to induce Th2 responses in the lung is unclear, but these cells might have more immunoregulatory properties. Altogether, the data suggest that trying to interfere with the functions of CD11b^+^ DCs might lead to new treatments for allergic asthma.

## T-cell subtypes and plasticity: which are relevant in the allergic phenotype?

### T-helper cell subsets: cytokine profiles

A seminal work from 1986 [27] suggested that the phenotypes of the Th cells could be separated into two forms: the Th1-cell secreting the cytokines IFN-γ and IL-12, whereas the Th2-cell secretes IL-4, IL-5 and IL-13. It soon became clear that primary cells from humans expressed a less clear dichotomy than the one observed for murine T-cell clones [28]. Nevertheless, the Th1-Th2 paradigm proved to be very productive for segregating the many observations on immunopathology. Most diseases of autoimmune origin were demonstrated to have a Th1 profile, while IgE-related diseases such as allergy and parasitic infestations were the prime example of a Th2-disease. In later years, several observations have emerged which rock the paradigm, the most prominent being various forms of regulatory T cells (Treg, Th3, Tr1) secreting mainly TGF-β and/or IL-10 [29], and the more recently described T cells secreting IL-17A and F, named Th17-cells [30]. The number of T-cell subsets defined on the basis of cytokine production has continued to grow with Th9 (IL-9-producing) and Th22 cells (IL-22-producing) as the latest newcomers. Also mixed forms, such as Th1/17 or Th2/22, have now been described, casting some doubt on the integrity of the individual Th-cell subtypes [31]. To a certain extent, the different cytokine production profiles of CD4+ cells seem to be mirrored by cytotoxic CD-8+ cells and recently even by the innate lymphoid cells [32].

### Relevant Th-subsets in allergy

The allergic immune response has been characterized by IgE, eosinophilia, and a T-cell response including the cytokines IL-4, IL-5, and Il-13. More recently, cytokines such as IL-9, IL-22, and IL-17 have also been implicated in the allergic inflammation. It is not yet clear, however, whether the allergic phenotype should include the CD4+ T-cell subsets being defined by these very cytokines: Th9, Th22, and Th17. Not all subtypes represent unique differential paths. Instead "cassettes" of stimulus–response-cytokine expression may co-exist in the same CD4+ T cell. One such "cassette" is seen with the IL-1 family of cytokines (IL-1beta, IL-18, and IL-33) which may modulate the expression of the cytokines such as IL-9 and IL-10 by other T-cell subsets [33,34]. Other such modulatory cytokines are TSLP and IL-25 [35,36], as well as some of the factors leading to the formation of the follicular T-helper cells that are believed to be important for the B-cell differentiation [37].

### DC-T cell interaction: the three-signal paradigm

**Figure 2:**
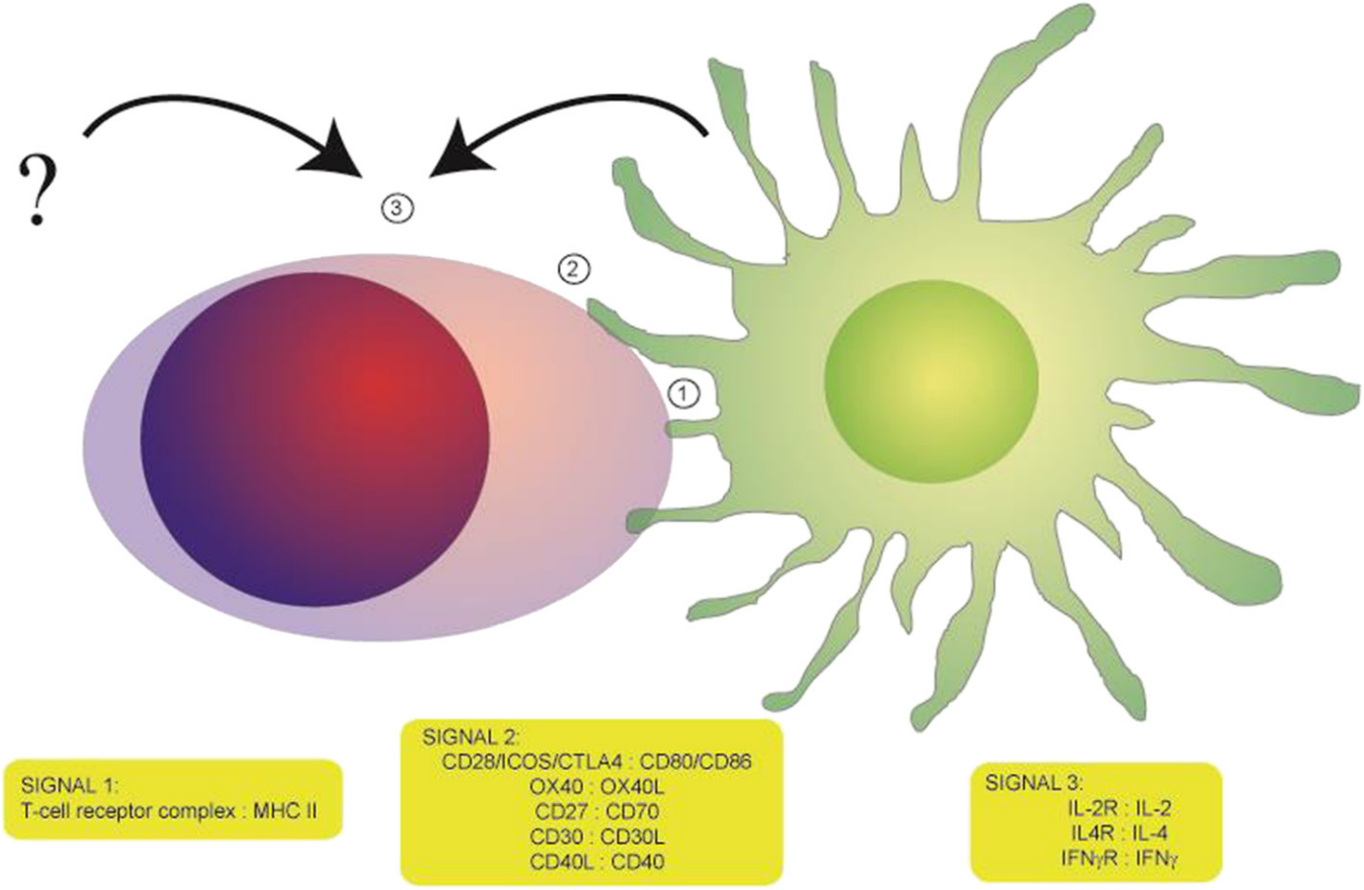
**The stimulation of the undifferentiated CD4+ T-helper cell by signals delivered to the T-cell receptor (Signal 1), by cell-to-cell-contact (Signal 2), and by soluble signals (Signal 3) from the dendritic cells or other adjacent cell types.** The signalling molecule pairs (with the relevant receptor on the T-cell mentioned first) are but a few examples of those described in the literature.

As described above for the lungs (Figure 1), different dendritic cell populations have the capability to act as MHC-bearing antigen-presenting cells for CD4+ T cells. Besides this primary signal (SIGNAL 1) that is recognized by the T-cell receptor, a number of other (antigen un-specific) signals are exchanged between the T cell, the dendritic cells, and perhaps other cells in the lymph node, leading to the differentiation of the T cell into different phenotypes. These signals comprise both cell-to-cell contacts (*i.e.*, mediated by pairs of cell surface-bound, co-stimulatory molecules) and soluble (cytokine:cytokine-receptor) interactions (Figure 2). The former comprises members of the immunoglobulin, the TNF-, and the TNF-receptor superfamilies [38], all of which include many soluble and cell-bound ligand-receptor pairs that are found throughout the immune system of both humans and rodents. As for many other cytokines and chemokines and their receptors, they appear to be of a certain promiscuity in the ligand-receptor preferences in the TNF-/TNFR superfamilies [39]. Signals delivered by surface-bound receptors, *e.g.*, CD80/CD86 to CD28, have been described as SIGNAL 2 and the soluble cytokines as SIGNAL 3, but the relative importance of the different costimulatory signals are still not clear (see Chen and Flies, 2013 [40] for a thorough discussion of co-stimulatory and co-inhibitory signals). What is important, however, is that the combination of co-stimulatory signals from the DC and perhaps from other cells is able to instruct the CD4 T-cell to differentiate. The main instructory signal for the formation of a Th2 cell is IL-4 [36], but the strength of the SIGNAL 1 seems to also influence whether a Th2 cell is formed. It is now clear that this differentiation is not just a choice between Th1 and Th2, but more subsets such as Th17, Treg, and Tfollicular helper (T_FH_) cells exist. Other subtypes, such as Th9 and Th22 may also exist, but it seems evident that there is a large degree of plasticity in forming the cytokine profile of a T cell. Thus, it is likely that the instruction of a T cell may be modular, in the sense that presence/absence of certain stimuli will instruct the T cell to produce different classes of cytokines.

### First induction of a Th2 response

A theme that has caused considerable discussion among immunologists is where the first IL-4 comes from. While the Th2 cell may produce sufficient IL-4 to develop and maintain the allergic inflammation and the IgE-synthesis, the finding that IL-4 is itself a differentiation factor of Th2-cells has suggested other cell types to be incriminated in the early events of Th2 development. A series of papers [41-43] indicated that basophils - that are known to secrete large amounts of IL-4 - could function as antigen presenting cells, but several later findings questioned such a capability due to the lack of close interaction with T-cells [44] and no or low expression of MHCII [45,46]. Also, suggestions of alternative explanations of the initial findings [24] weakened the case for basophils as a primary inducer of Th2 responses, even though the IL-4 contribution from this cell may still be important in creating the necessary cytokine environment for a Th2-shift. Other cell types such as innate lymphoid cells (ILCs) have also been suggested as being very important for the induction of a Th2 response. While the first reports on these cell types came before the year 2000, only recently has this area expanded tremendously (reviewed in [47]) with the discovery of several subtypes (based on cytokine expression) such as ILC1, ILC2 [48], etc., in parallel with the findings from the cognate immune response, where both CD4+ and CD8+ T-cells are classified the same way. ILCs do not express T-cell receptors, but can be activated by a large number of other stimuli. For the ILC2, it is interesting that they seem to be stimulated by IL-25, IL-33 and TSLP, all cytokines that have been connected with the early phases of Th2 induction. While these cells have been incriminated in both asthmatic and airway hyper-reactivity responses and in mounting inflammation in helminth infections, there is still no consensus as to whether they may actually drive the cognate Th2-response.

### T-cell subsets: migratory profiles and organ specificity

T cells may also be subdivided based on their migrating capabilities. Many subsets such as central memory (T_CM_), follicular memory (T_FM_), and T_FH_ have been described based on their migration in the lymphoid system. Also, organ specific subsets from the gut (expressing the chemokine receptor CCR9 and the α4β7-integrin adhesion molecule) or the skin (expressing CLA, CCR10, and CCR4) have been described.

Depending on the manifestation of clinical symptoms in different organs such as the gut, the skin, or the airways, T cells may often be found in each of these inflammatory foci with different characteristics. Whether these cells are the primary drivers of the disease or secondary to the primary sensitization is not known, but the generalized IgE-immune response most often seen in food allergies could suggest that the initiation of the food allergic immune reaction is not always related to the organ in which elicitation takes place. In this respect, it is interesting that recent studies of food allergy applying the tetramer technique for enumerating allergen specific T cells have found relatively few gut-specific (α4β7+) T cells compared to a higher frequency of skin-specific (CLA+) T cells [49], although varying results have been found by others [50]. Future studies will have to further address the role of the allergen-specific CD4+ T cells and their localization. It is likely that such studies will help to increase understanding of the sensitization process, which may ultimately lead to better primary prevention of food allergy.

## B-cell isotype switch and differentiation into IgE-producing plasma cells

### Long-lived plasma cells

A key event in the pathogenesis of allergy is the production of IgE antibodies. Antibodies are secreted by plasma cells and their precursors, the plasma blasts. For many years, plasma cells have been considered as short-lived end-stage products of B-cell differentiation; however, current studies argue for the presence of long-lived resident plasma cells that are located in survival niches primarily found in the bone marrow and inflamed tissue [51]. Once settled, long-lived plasma cells are difficult to target therapeutically because they are resistant to immunosuppression and irradiation [52,53]. More knowledge on preventable causes of IgE plasma cell development is therefore required.

### Isotype switching

To become an IgE-producing plasma cell, the B cell needs to isotype switch to IgE. This process is induced by two signals provided by a Th2 cell. The first signal is delivered by the cytokines IL-4 or IL-13, which target the Cϵ gene for initiating switch recombination. The second signal is delivered by interaction of the cell surface antigen CD40 with its ligand (CD40L) expressed on activated T cells. Once the IgE-positive B cells are formed, they are able to differentiate into non-dividing, IgE-producing plasma cells. Although some populations of long-lived plasma cells persist in the spleen, most of them return to the bone marrow or invade inflamed tissues, where they survive up to several months or even a lifetime in survival niches as resident, immobile cells [54].

### *In vitro* B-cell model

To study mechanisms controlling the B cell, it is advantageous to have a well-defined *in vitro* model. A B-cell assay has been developed, which supports the survival and differentiation of naïve B cells to IgE-producing plasma cells [55]. The kinetic expression of a broad panel of B-cell markers, immunoglobulins, and activation factors has been determined over 12 days of stimulation with IL-4 and anti-CD40.

**Figure 3:**
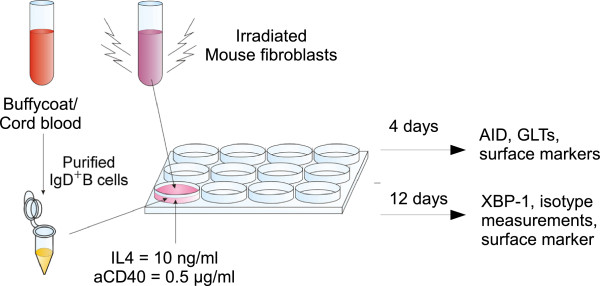
In vitro B-cell model.

The B-cell model (Figure 3) was used for investigating the difference in isotype switch and plasma cell differentiation in B cells isolated from adult blood compared to cord blood. IgD-positive B cells were purified from buffycoat or cord blood-isolated peripheral blood mononuclear cells (PBMCs) using mouse anti-human IgD coupled to magnetic beads. B cells were stimulated with IL-4 + anti-CD40. FcγRII/CD32 transfected mouse fibroblasts were used to stabilise the anti-CD40 presentation. After 4 days of stimulation, the early class switch recombination markers activation-induced deaminase (AID) and germline transcripts (GLTs) were measured by real-time PCR and traditional PCR, respectively. After 12 days of stimulation, the plasma cell marker XBP-1 was measured by real-time PCR, and immunoglobulins were measured in supernatants by ELISA. Surface markers were determined at various times by flow cytometry. IgD-positive B cells from healthy nonallergic donors did not spontaneously secrete IgE in the culture supernatants. It was shown that several germline immunoglobulin genes are constitutively transcribed in adult naïve human B-cell populations, and that IL-4 and anti-CD40 antibody enhance the transcription of not only ϵ and γ4 GLT but also γ2 and γ3 with a maximal expression at day 6. This subsequently leads to Ig-production which can be determined on the cell surface from day 6 and in the cultures as IgE, IgG4, and total IgG clearly increasing from day 8 and onwards. AID was used as a marker to identify preswitching B cells, and was found to be highly up-regulated after three days of stimulation. Finally, it was shown that after eight days of stimulation, the B cells develop into plasma cells phenotypically defined as CD138^+^intracellular IgE^+^ cells. Cord blood B cells were able to isotype switch to IgE at a comparable level to adult B cells. However, contrary to the adult B cells, cord blood B cells were able to differentiate into CD138^+^intracellular IgE^+^ plasma cells to a much higher level. The levels of secreted IgE and IgG4 were found to be comparable to adult B cells [56].

A well-defined B-cell model has been developed, which is capable of producing high levels of IgE allowing investigation of regulators affecting the induction of IgE-producing plasma cells. It has not been tested if the model could produce specific IgE, and this limitation should be kept in mind. The stimulation via CD40 and the IL-4 receptor initiates a programmed sequence of events transforming B cells to IgE and IgG4-producing plasma cells in both cord blood and adult peripheral blood. However, the younger nature of cord blood B cells seems to be more potent in differentiating into IgE-producing plasma cells compared to adult B cells.

## Discussion

Allergic sensitization is the outcome of a complex interplay between allergen and host in a given environmental context. Certainly, not every molecule will have the properties to induce sensitization, either lacking the required intrinsic pro-allergenic properties or being presented in the wrong context (*e.g.*, quantity, time of exposure, co-exposures). Here, the authors have highlighted that the process of sensitization is orchestrated by a combination of communicating structural cells and innate and adaptive immune cells. Understanding the immunological and biochemical mechanisms underlying the process of sensitization is of the utmost importance to explain why a certain exposure leads to allergic sensitization. The genetic background and epigenetic make-up of the host shape the way it will respond to contact to a potential allergen, *i.e.*, whether the host will become tolerant, sensitized, and/or allergic. At all subsequent encounters of a potential allergen with different cell subsets during its passage over the mucosal barrier, the (epi)genetic background of the host, in combination with the full spectrum of environmental co-exposures (*e.g.*, infections, pollution) and the timing of exposure, will determine the outcome. Sensitizing properties of proteins can therefore never be seen in isolation.

## Competing interests

The authors declare that they have no competing interests.

## Authors’ contributions

RvR, LH, MP, LKP, and ES were speakers at the April 2012 Symposium on Sensitizing Properties of Proteins and contributed written summaries of their presentations to this manuscript. All authors read and approved the final manuscript.
